# Abiraterone acetate plus prednisone for the Management of Metastatic Castration-Resistant Prostate Cancer (mCRPC) without prior use of chemotherapy: report from a large, international, real-world retrospective cohort study

**DOI:** 10.1186/s12885-019-5280-6

**Published:** 2019-01-14

**Authors:** Martin Boegemann, Sara Khaksar, Guillaume Bera, Alison Birtle, Catherine Dopchie, Louis-Marie Dourthe, Els Everaert, Martin Hatzinger, Dirko Hercher, Werner Hilgers, Geoffrey Matus, Laura Garcia Alvarez, Laurent Antoni, Martin Lukac, Geneviève Pissart, Paul Robinson, Tony Elliott

**Affiliations:** 1 Department of Urology, University of Muenster Medical Center, Albert-Schweitzer-Campus 1, GB A1, D-48149 Muenster, Germany; 2 St Luke’s Cancer Centre, The Royal Surrey County Hospital, Guildford, UK; 3 Groupe Hospitalier Bretagne Sud, Hôpital du Scorff, Lorient, France; 4 Rosemere Cancer Centre, Royal Preston Hospital & University of Manchester, Manchester, UK; 5 CHWapi site IMC, Tournai, Belgium; 6 Clinique Saint Anne, Strasbourg, France; 7 AZ Nikolaas, Sint Niklaas, Belgium; 8 Agaplesion Markus Hospital, Frankfurt, Germany; 9 Refrath Urological Center, Bergisch Gladbach, Germany; 10 Institut Sainte Catherine, Avignon, France; 11 CHC Cliniques Saint Joseph, Liège, Belgium; 12 IQVIA, Barcelona, Spain; 13 Janssen Pharmaceutica NV, Beerse, Belgium; 14 PAREXEL International, Prague, Czech Republic; 15 Janssen EMEA, London, High Wycombe UK; 16 The Christie Hospital, Manchester, UK

**Keywords:** Metastatic castration-resistant prostate cancer, Time to treatment failure, Abiraterone acetate, Real-world evidence

## Abstract

**Background:**

With the recent introduction of novel treatment options, real-world data from patients with metastatic castration-resistant prostate cancer (mCRPC) are required to better understand the impact on routine clinical practice. This study primarily aimed to describe the time to treatment failure (TTF) of mCRPC patients treated with abiraterone acetate plus prednisone or the corticosteroid of choice (AAP) in the pre-chemotherapy setting. Other relevant outcomes, clinical and treatment characteristics of these patients were also evaluated.

**Methods:**

This retrospective, observational study collected data from chemotherapy-naïve mCRPC patients treated with AAP from four European countries. Kaplan-Meier curves were used to estimate TTF, progression-free survival (PFS), and time to first skeletal-related event. The impact of baseline characteristics on TTF and PFS was explored using univariate and multivariate Cox proportional hazard models. Log-rank test was used to assess the potential role of duration of response to ADT in predicting response to AAP treatment.

**Results:**

Data from 481 eligible patients (Belgium: 68; France: 61; Germany: 150; UK: 202) were analysed. At AAP initiation, the median age of patients was 75.0 years (interquartile range [IQR]: 69.0–81.0), and the median PSA was 56.2 ng/mL (IQR: 22.2–133.1), with over 50% of patients presenting an ECOG score of 0 or 1. Visceral metastases were present in 7.5% of patients; an exclusion criterion in the COU-AA-302 clinical trial. The median TTF with AAP was 10.0 months (95%CI: 9.2–11.1) and the median PFS was 10.8 months (95%CI: 9.6–11.8). Shorter TTF was significantly associated with higher ALP (> 119 units/L), higher PSA (> 56.2 ng/mL), or poorer ECOG PS scores at AAP initiation (*p* < 0.05). Patients with longer duration of response to ADT (≥12 months) presented longer TTF and longer time to progression (*p* < 0.0001).

**Conclusions:**

This European real-world study provides valuable insights into the characteristics, treatment, and outcomes of chemotherapy-naïve patients with mCRPC who received AAP in routine clinical practice. Treatment effectiveness of AAP in the real-world is maintained despite patients having poorer clinical features at initiation than those observed in the COU-AA-302 trial population.

**Electronic supplementary material:**

The online version of this article (10.1186/s12885-019-5280-6) contains supplementary material, which is available to authorized users.

## Background

In recent years, new treatments for metastatic castration resistant prostate cancer (mCRPC) have become available, including abiraterone acetate (Zytiga®) plus prednisone (AAP). A phase III pivotal study of chemotherapy-naïve mCRPC patients (COU-AA-302) [1, 2] demonstrated improvements in median radiographic progression-free survival (rPFS) from 8 to 16 months [1] and median overall survival (OS) from 30.3 to 34.7 months [2] with AAP as compared with prednisone plus placebo. The median duration of AAP treatment was 13.8 months [1, 2]. Based upon these data, AAP was approved by the European Medicines Agency in November 2012 for the treatment of mCRPC in patients who are asymptomatic or mildly symptomatic after the failure of ADT, and in whom chemotherapy is not yet clinically indicated [3].

Healthcare authorities and budget holders are increasingly requesting real-world data to advance knowledge of the relative effectiveness and value of a new treatment in the management of patients in routine practice. Information on time to treatment failure and other clinical outcomes from the real-world setting are important to support and/or supplement the evidence generated from randomized control trials (RCTs). Real-world data from well-designed studies provide valuable information across the full spectrum of patients who are eligible to be treated in routine practice, in addition to the narrower population who meet the eligibility criteria in RCTs. The majority of mCRPC patients treated in clinical practice are elderly, and comorbidities such as cardiovascular disease, hypertension and diabetes mellitus are thus common. Such patients along with those with visceral metastases may be under-represented in RCTs of mCRPC [1, 2]. At present, there are no defined predictive factors of treatment response in mCRPC management. However, the potential value that different clinical parameters may have for physicians to better understand response to treatment in these patients has been previously investigated. Loriot et al. [4] reported that a shorter duration of response to ADT (< 12 months) was significantly associated to shorter PFS and lower PSA response (defined as a decline of serum PSA level ≥ 50%) in patients treated with AAP or other androgen receptor axis targeted drugs. Thus, further investigation may be warranted to better understand the role of these clinical parameters in the management of mCRPC patients in the real-world.

The main objective of this retrospective observational study was to add to the body of knowledge related to abiraterone acetate treatment by primarily describing the time to treatment failure of chemotherapy-naïve mCRPC patients treated with AAP in routine clinical practice in Europe. Other relevant outcomes, clinical and treatment characteristics of these patients were also evaluated. In addition, subgroup analyses of patients based on their baseline characteristics were also undertaken to further explore the potential role that pragmatic clinical parameters may play in predicting treatment response in these patients.

## Methods

### Study design and eligibility

This was a retrospective, observational cohort study conducted at multiple sites in Belgium, France, Germany and the United Kingdom (UK). The data collection period for each patient ranged from the initial date of prostate cancer diagnosis up to the date of data collection. The start of AAP treatment was considered as baseline. Data from eligible patients treated with AAP were extracted from their medical records and entered into electronic case report forms (eCRF).

An initial group of physicians was approached to evaluate their interest to participate; those interested were asked to complete a feasibility questionnaire to further assess their suitability for the study in terms of possible number of patients they could enrol, experience in prior studies, data availability and contracting/ethic approval timelines. Subsequently, decisions on which sites to enrol were made based on their potential of each physician to provide patients and their ability / experience to complete the study within timelines. High prescribers were not specifically targeted or selected during this exercise.

The participating physicians were specialists in urology and oncology who routinely managed patients with advanced prostate cancer, and were based in a range of hospitals, clinics, and office-based centres. At each site, participating physicians were asked to check for eligible patients starting with patients who initiated AAP treatment from 1 January 2013 (or the date that AAP first became available at the site) up to 31 December 2014, and consecutively include patients in the study in the order in which they were assessed for eligibility. This strategy was used in order to minimise any potential selection bias of patients enrolled into the study. Patient data was collected between November 2015 and October 2016. All patients were treated with abiraterone acetate in combination with corticosteroid according to routine clinical practice at each site.

Where applicable, the study protocol was reviewed by an Independent Ethics Committee (IEC) or Institutional Review Board (IRB). In addition, the study was presented or notified to regional and site IEC/IRBs if required by local laws or regulations or hospital policies. Specifically: i) in Belgium, each of the IECs from all the participating sites approved the study (ie, Comité d’Ethique - Erasme ULB, Comité d’Ethique CHWAPI, Comité d’Ethique Centre Hospitalier Peltzer-La Tourelle, Commissie voor Medische Ethiek St. Jozefkliniek Izegem, Commissie voor Medisch Ethiek AZ Groeninge Kortrijk, Commissie voor Medisch Ethiek AZ Klina, Ethisch Comité AZ Nikolaas, Comité d’Ethique Médical CHC Saint Joseph Liège, Commissie voor Ethiek AZ St. Jan Brugge Oostende); ii) in France, national approvals were granted by CCTIRS, CNOM and CNIL; iii) in Germany, the IEC committee granting approval was the “Ethik-Kommission der Ärztekammer Westfalen-Lippe und der Westfälischen Wilhelms-Universität” (reference number: 2015–526-f-S), belonging to the Medical Council Westfalen-Lippe; and iv) in the UK the research ethics committee (REC) providing approval was the “London – South East REC” (reference number: 16/LO/0232). Patients who were still living also had to give written informed consent in accordance with local requirements; deceased patients who met the eligibility criteria were enrolled automatically.

Patients were eligible if they had documented mCRPC and had received AAP for the treatment of mCRPC that was asymptomatic or mildly symptomatic (per physician’s evaluation) after failure of ADT at the time of treatment initiation. Patients were excluded if they had received any chemotherapy or cytotoxic agent for treatment of mCRPC before initiation of AAP, were treated with AAP as part of an interventional study, or received any investigational treatment either previously or simultaneously with AAP.

### Data collection and outcomes of interest

Demographic and clinical characteristics at the time of AAP treatment initiation and discontinuation were collected. These included age, comorbidities, key biochemical parameters (baseline prostate-specific antigen [PSA], hemoglobin, alkaline phosphatase [ALP] and lactate dehydrogenase [LDH] levels), Eastern Cooperative Oncology Group Performance Status (ECOG-PS) and Gleason score.

The main endpoint of the study was time to treatment failure with AAP. Time to treatment failure was defined as time from start of AAP treatment to treatment discontinuation for any reason, including disease progression, intolerance of treatment, or death from any cause, and was considered equivalent to treatment duration. Secondary endpoints included progression-free survival (PFS), survival rate at one year, and time to first skeletal-related event. Data on treatments received after discontinuation of AAP was also collected.

PFS was defined as time from start of AAP treatment to progression or death from any cause. Progression was assessed by physician evaluation (based on routine care information in the medical record) of three endpoints: PSA progression; radiographic progression (radiographic evidence of new metastasis and/or tumour spreading as measured through bone, computerized tomography [CT] and/or magnetic resonance imaging [MRI] scans); or symptomatic/clinical progression (patient record confirmed one or more of the following: increased pain, skeletal related events, increased requirement for analgesics, or need for palliative radiotherapy). No further evaluations for the ascertainment of progression were required to the physician’s besides what is described above. Skeletal related events included pathological fracture, spinal cord compression, radiotherapy or surgery to the bone.

All adverse drug reactions (ADRs) following exposure to AAP, whether serious or non-serious, were recorded if they were confirmed in the source documentation to be at least possibly related to AAP. Selected concomitant medications during the treatment with AAP were also collected retrospectively.

### Statistical analysis

All patients who met the eligibility criteria were included in the data set for analysis. The objective of the study was primarily descriptive, and most of the outcomes were analysed using descriptive statistics (for categorical variables number and percentage of patients per response option, based on non-missing data; for continuous variables, the median and the inter-quartile range [IQR] are reported). Time-to-event endpoints were analysed using Kaplan-Meier survival plots. For all time-to-event endpoints, patients who had not experienced the event of interest at the time of data collection were censored. Missing data were not imputed.

The impact of covariates on time to treatment failure and PFS was explored using univariate and multivariate Cox proportional hazard models. Baseline variables included in the multivariate models were defined based on statistical significance in univariate analysis (*p*-value < 0.10). For laboratory parameters, the median of the study patient population was used as the cut-off to categorise variables for inclusion into the model. Use of categorical variables rather than continuous variables was selected to control for the impact of extreme values in a population where there are not ranges for normal laboratory values, as well as to maximise the statistical power defining two groups with the same number of patients. Final variables associations were reported as hazard ratios (HR) with 95% CI and corresponding *p*-values.

Subgroup analyses using the log rank test were undertaken to evaluate the impact of sensitivity to prior ADT treatment on AAP treatment duration; to that end, time to treatment failure of patients with a longer period between castration and mCRPC diagnosis (≥12 months) was compared to those patients with a shorter period (< 12 months). In addition, the baseline characteristics of those patients with longer time to treatment failure with AAP (ie, upper quartile) were also compared to the rest of the patients using univariate and multivariate regression models.

Due to the lack of formal hypotheses tested and the primary descriptive purpose of the study, sample size calculation was based on precision estimation. The precision around the primary endpoint (time to treatment failure with AAP) was estimated based on data reported in the Phase III COU-AA-302 trial [1, 2]. It was anticipated that a sample size of 540 patients across the four countries would allow for 95% confidence intervals (CI) around the main endpoint of time to treatment failure below ±1.48, based on a mean AAP treatment duration of 13.8 months, an estimated standard deviation (SD) of 17.5 months, and assuming a normal distribution.

## Results

A total of 481 patients were recruited and eligible from across Belgium (*n* = 68), France (*n* = 61), Germany (*n* = 150), and the UK (*n* = 202); a further 43 patients were recruited but excluded for non-eligibility. Of the 43 non-eligible patients, a total of 16 patients met an exclusion criterion: 13 patients had received a chemotherapy/cytotoxic agent to treat their mCRPC before AAP initiation, two patients had been treated with AAP as part of an interventional study, and one patient had received an investigational treatment for prostate cancer of any kind before or simultaneously with AAP. The rest of the non-eligible patients were excluded due to violations of different inclusion criteria or for other reasons (e.g., physician withdrawal, source data not available). Three patients did not have histologically or cytologically confirmed diagnosis of adenocarcinoma of the prostate, three patients did not have documented metastatic prostate cancer, one patient did not have documented castration resistance with progression of prostate cancer on ADT, three patients had received AAP treatment prior to mCRPC diagnosis, three patients initiated AAP after 31st December 2014, and three did not have written informed consent. Eleven patients were excluded for other reasons. The final sample size of 481 patients provided a level of precision (95% CI) of ±0.8 months around the estimate of time to treatment failure, which represented a better level of precision than initially estimated. The median duration of follow-up from AAP initiation was 18.8 months (IQR: 11.9–25.1) for the whole group, and 23.4 months for the alive patients (IQR: 19.2–29.1).

### Patient characteristics at AAP initiation

The median age of patients at initiation of AAP was 75.0 years, with only 11.0% of patients aged < 65 years. Demographic and clinical characteristics at initiation of AAP are shown in Table 1.

**Table 1:** Demographic and clinical characteristics at AAP initiation (baseline)

Variable	All patients (*N* = 481)
Age (years)	
Median (IQR)	75.0 (69.0–81.0)
< 65, N (%)	53 (11.0)
65–74, N (%)	168 (34.9)
≥ 75, N (%)	260 (54.1)
Comorbidities, N (%)^a^	
No comorbidities reported	152 (31.6)
At least one comorbidity reported	329 (68.4)
*Any Cardiovascular event*	281 (58.4)
Hypertension	214 (44.5)
Angina pectoris	27 (5.6)
Myocardial infarction	24 (5.0)
Arrhythmia	45 (9.4)
Cerebrovascular accident	12 (2.5)
Transient ischemic attack	8 (1.7)
Other cardiovascular event	59 (12.3)
*Thromboembolic disease*	10 (2.1)
*Any renal disorders*	52 (10.8)
*Diabetes mellitus*^*b*^	71 (14.8)
ECOG performance status, N (%)	
Unknown	202 (42.0)
0	110 (22.9)
1	134 (27.9)
2	31 (6.4)
3–4	4 (0.8)
Gleason score at initial prostate cancer diagnosis, N (%)	
Unknown	46 (9.6)
≤ 7	204 (42.4)
≥ 8	231 (48.0)
Laboratory parameters, median (IQR)	
PSA (ng/mL, *N* = 456)	56.2 (22.2–133.1)
Alkaline phosphatase (units/L, N = 357)	119.0 (81.0–231.0)
Lactate dehydrogenase (units/L, *N* = 184)	277.0 (197.5–382.5)
Hemoglobin (g/dL, *N* = 375)	12.5 (10.8–13.8)
Location of metastases, N (%)	
Bone and/or lymph node metastases	429 (89.2)
• Bone	378 (78.3)
• Non-regional lymph node/s	63 (13.1)
• Reginal lymph node/s	201 (41.8)
Visceral (non-nodal soft tissue)	36 (7.5)
Non-categorised	16 (3.3)

^a^List of individual comorbidities is not mutually exclusive (same patient may have had two or more comorbidties). ^b^Includes insulin-dependent and non-insulin dependent. Abbreviations: *ECOG* Eastern Cooperative Oncology Group, *IQR* interquartile range, *PSA* prostate-specific antigen

At initial prostate cancer diagnosis, 48% of patients had Gleason scores of 8–10, and 42% had Gleason score of 7 or less (scores for the remainder were unknown). A total of 139 patients (28.9%) presented distant metastasis at the time that prostate cancer was initially diagnosed, with 25.2% of patients for whom data on distant metastasis was unknown (not reported on the patient records).

ECOG PS at AAP initiation was unknown in 42% of patients. Of those for whom it was reported, most (87%) had ECOG PS 0 or 1. Patients presented with a median PSA level of 56.2 ng/mL at the start of treatment. There was a relatively large proportion of patients suffering from cardiovascular diseases (58.4%), diabetes (14.8%) and renal disorders (10.8%). The most common sites of metastasis were bone and lymph node (89.2%), but 7.5% of patients had visceral metastases, which were excluded from the phase III COU-AA-302 trial. Liver metastases were present in 11 of the 36 patients with visceral metastases. The large majority of patients (93.6%) had received ADT treatment rather than undergoing a bilateral orchiectomy.

Progression on ADT was cited as a reason for initiation of AAP in 100% of patients. PSA progression was reported for 83.8% of patients, radiographic progression for 50.5% of patients, and symptomatic progression for only a minority of patients (14.8%).

### Abiraterone acetate treatment

As per SmPC [5], abiraterone acetate was administered together with corticosteroids in all patients; typically prednisone or prednisolone, although in some cases dexamethasone was used. Accordingly, in this study, the commonly used term “AAP” refers to abiraterone acetate plus the corticosteroid of choice. The median time from initial diagnosis of prostate cancer until initiation of AAP treatment was 5.0 years (IQR: 2.0–9.0 years). All but two patients were initiated on AAP at the recommended dose of 1000 mg/day, and dose remained unchanged for 96% of patients.

**Fig. 1:**
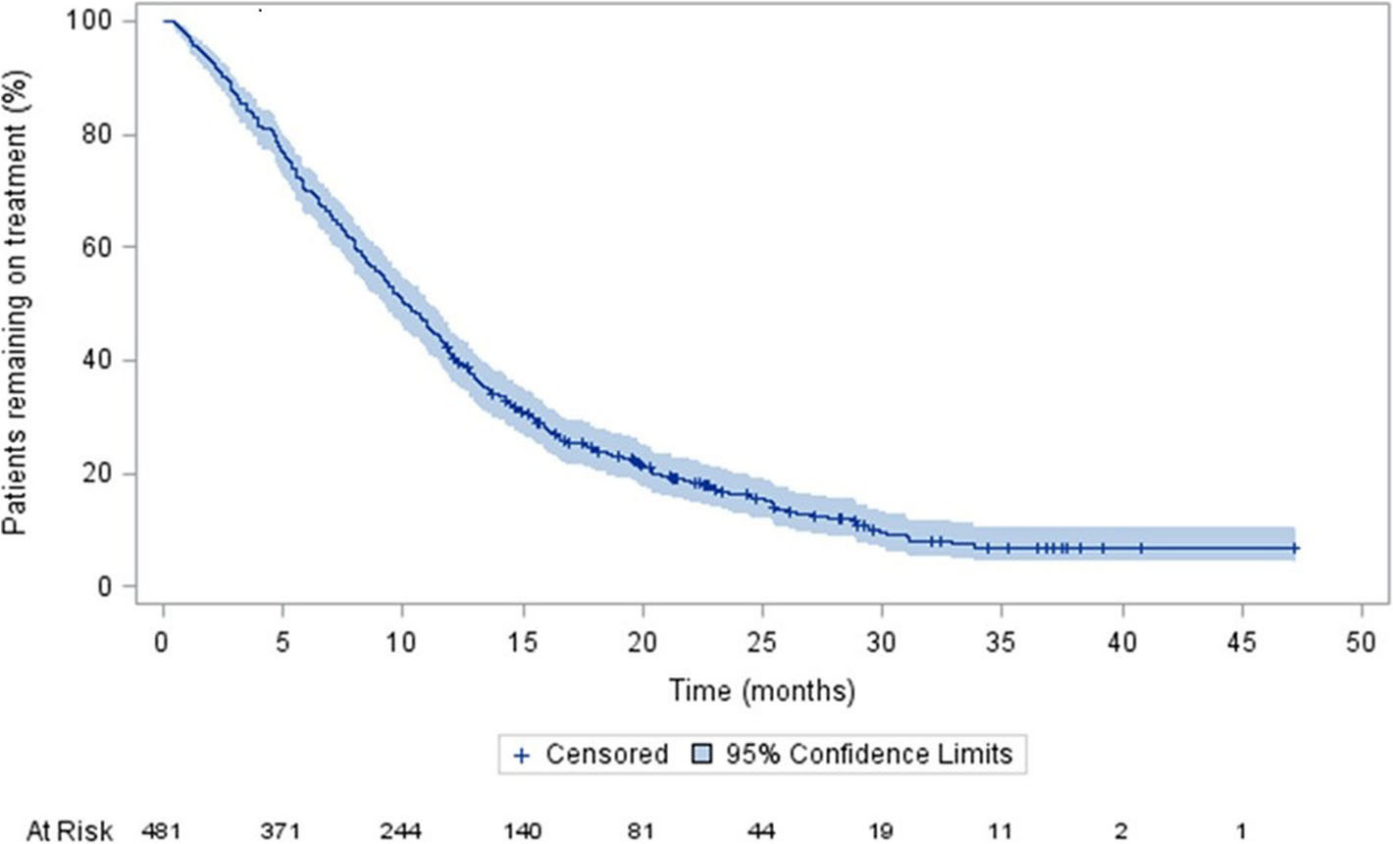
Time to treatment failure with AAP

The median time to treatment failure with AAP was 10.0 months (95% CI: 9.2–11.1) (Fig. 1). In total, 412 patients (85.7%) discontinued treatment during the follow-up period. Importantly, with respect to the retrospective study design and primary objective, sixty-nine patients (14.3%) were continuing on treatment with AAP at the point of data collection; these patients were censored and the study design did not allow for further follow-up. Of those who discontinued, 55 (11.4% of study patients) were reported as discontinuing due to death; none of the deaths was considered to be related to AAP.

Disease progression (PSA, radiographic, clinical or any combination) was the main reason for discontinuation of AAP, given as a reason in 328 (68.2%) of study patients. A substantial proportion of these patients (*n* = 97, 29.6%) had PSA progression reported as the only reason for discontinuation. A further 10.7% had symptomatic progression as the only reason for discontinuation, and 8.8% had radiographic progression only. Discontinuation due to toxicity was reported in only 1.9% of patients. The remainder of patients who discontinued had the reason reported as ‘other’. Patients who progressed based on a combination of PSA, symptomatic and radiographic factors represented 10.1%.

#### Subgroup analyses

In order to assess whether sensitivity to prior ADT treatment or selected clinical characteristics can impact duration of AAP treatment or disease progression, the differences on time to treatment failure with AAP and time to progression among selected patient subgroups were evaluated. Patients with 12 or more months between bilateral orchiectomy or ADT treatment and mCRPC diagnosis had longer time to treatment failure (Table 2) and longer time to progression estimates than those with less than 12 months.

**Table 2:** Time to treatment failure on AAP across different patient subgroups based on the period of time between castration and mCRPC diagnosis

Time between bilateral orchiectomy or ADT treatment and mCRPC diagnosis	Percentile	Estimate of Time to Treatment Failure on AAP (months) (95% CI)	Logrank Test *p*-value
< 12 months (*n* = 87)	75	11.9 (10.0–14.0)	< 0.0001
	50	7.9 (6.6–8.9)	
	25	4.4 (2.3–5.8)	
≥12 months (*n* = 338)	75	19.7 (16.3–21.2)	
	50	11.5 (10.0–12.1)	
	25	5.6 (5.0–6.8)	

### Other outcomes

**Fig. 2:**
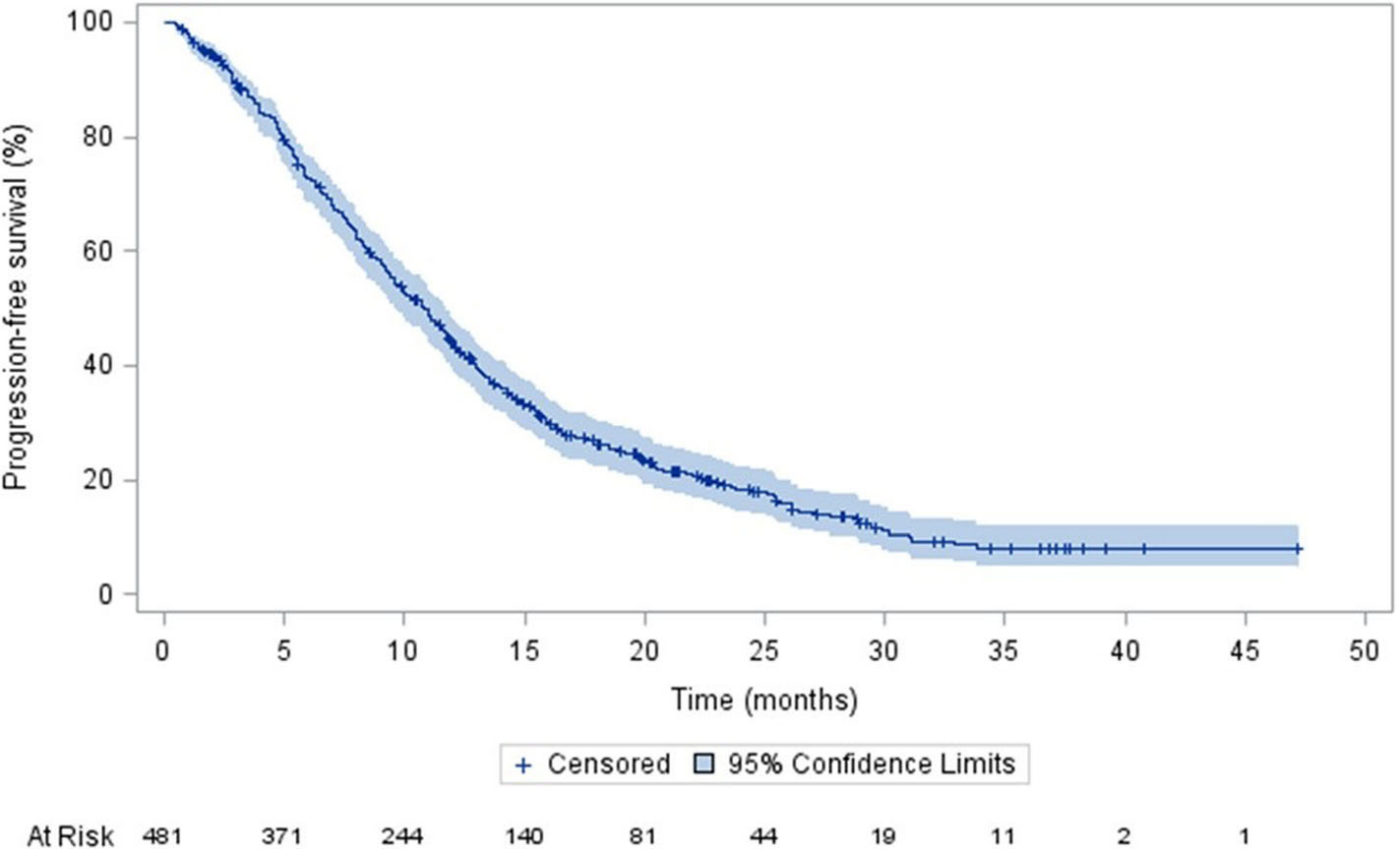
Progression-free survival of mCRPC patients treated with AAP

Median PFS on AAP treatment was 10.8 months (95% CI: 9.6, 11.8; Fig. 2). Given that treatment discontinuation was mainly due to disease progression, time to treatment discontinuation and time to progression is the same for most patients.

The one-year survival rate was 74.8%. Only 55 patients had a skeletal-related event recorded after initiation of AAP, and the median time to first skeletal-related event was not reached. The proportion of patients with a skeletal-related event reported was 8.9, 11.0 and 11.5% at 12, 24 and 36 months, respectively. The proportion of patients with skeletal-related events, exposed and non-exposed to bone protective agents, was 15.7 and 8.7%, respectively.

### Treatments after discontinuation of AAP

At the time of data collection, 73.1% of patients who discontinued AAP for reasons other than death (*n* = 357) were reported to receive treatments after AAP discontinuation (*n* = 261), and specific therapies were reported by investigators for all but seven of these patients (Additional file 1: Table S1).

Approximately half (51.6%) of the 254 patients for whom a therapy was recorded received chemotherapy or cytotoxic agents as first treatment after AAP discontinuation. This was almost always taxanes (docetaxel or cabazitaxel); less than 1% of patients received other cytotoxic drugs. Median time to initiation of first chemotherapy treatment after discontinuation of AAP was 3.3 months (95% CI: 2.2 to not reached).

The second most common therapy after discontinuation of AAP was enzalutamide (24.8% of patients for whom a therapy was recorded). Various other treatments were prescribed as first choice after AAP discontinuation. Hormonal therapy alone was used in 11.8%, of those for whom a therapy was recorded, while 7.9% were treated with radium-223 dichloride.

### Factors influencing time to treatment failure with AAP

In univariate analysis of the relationship between clinical characteristics at initiation and time to treatment failure, higher ALP (> 119 units/L), or higher LDH (> 277 units/L), or higher PSA (> 56.2 ng/mL), or higher Gleason score (≥ 8) or poorer ECOG PS scores were all associated with shorter time to treatment failure with AAP (*p* < 0.05). Higher hemoglobin (> 12.5 g/dL) was associated with longer time to treatment failure (p < 0.05) (Table 3).

**Table 3:** Univariate and multivariate analysis (Cox regression models) assessing the relationship between baseline characteristics and time to treatment failure with AAP

Covariate	Frequency	Univariate HR (95% CI) *p*-value^1^	Multivariate: no selection HR (95% CI) *p*-value^2^	Multivariate: backward selection HR (95% CI) *p*-value^3^
Age				
Unknown	0	N/A	N/A	
< 65	53	Reference	Reference	
65–75	188	1.011 (0.721–1.416) *p*-value = 0.9516	1.008 (0.711–1.429) *p*-value = 0.9651	
> 75	240	1.230 (0.886–1.708) *p*-value = 0.2154	1.148 (0.808–1.632) *p*-value = 0.4414	
Comorbidities				
Unknown	0	N/A	N/A	
0	152	Reference	Reference	
1	143	1.253 (0.980–1.603) *p*-value = 0.0719	1.288 (0.994–1.669) *p*-value = 0.0556	
2	89	1.148 (0.865–1.525) *p*-value = 0.3392	1.140 (0.845–1.540) *p*-value = 0.3909	
≥ 3	97	1.070 (0.805–1.422) *p*-value = 0.6413	0.937 (0.685–1.281) *p*-value = 0.6844	
Alanine aminotransferase (ALT)				
Unknown	127	1.024 (0.803–1.304) *p*-value = 0.8498	0.969 (0.696–1.350) *p*-value = 0.8534	
≤ 18	184	Reference	Reference	
> 18	170	0.825 (0.658–1.035) *p*-value = 0.0960	0.840 (0.657–1.073) *p*-value = 0.1623	
Alkaline phosphatase (ALP)				
Unknown	124	1.111 (0.861–1.435) *p*-value = 0.4175	0.879 (0.624–1.239) *p*-value = 0.4627	0.964 (0.718–1.296) *p*-value = 0.8091
≤ 119	182	Reference	Reference	Reference
> 119	175	1.816* (1.451–2.273) *p*-value = < 0.0001	1.491* (1.160 - 1.915) *p*-value = 0.0018	1.510* (1.189–1.919) *p*-value = 0.0007
Aspartate aminotransferase (AST)				
Unknown	248	1.294 (1.017–1.645) *p*-value = 0.0358	1.049 (0.758–1.452) *p*-value = 0.7709	
≤ 24	118	Reference	Reference	
> 24	115	1.267 (0.953–1.684) *p*-value = 0.1034	1.183 (0.861–1.625) *p*-value = 0.3005	
Hemoglobin				
Unknown	106	0.850 (0.661–1.095) *p*-value = 0.2085	1.039 (0.746–1.448) *p*-value = 0.8196	1.064 (0.785–1.443) *p*-value = 0.6890
≤ 12.5	192	Reference	Reference	Reference
> 12.5	183	0.666* (0.534 - 0.831) *p*-value = 0.0003	0.783* (0.618–0.991) *p*-value = 0.0414	0.779* (0.621–0.977) *p*-value = 0.0309
LDH				
Unknown	297	1.294 (0.996–1.681) *p*-value = 0.0536	1.228 (0.867–1.740) *p*-value = 0.2467	
≤ 277	92	Reference	Reference	
> 277	92	1.546* (1.124–2.125) *p*-value = 0.0074	1.278 (0.904–1.806) *p*-value = 0.2467	
PSA				
Unknown	25	1.540 (0.997–2.379) *p*-value = 0.0518	1.674 (1.053–2.663) *p*-value = 0.0295	1.593 (1.012–2.506) *p*-value = 0.0442
≤ 56.2	228	Reference	Reference	Reference
> 56.2	228	1.663* (1.359 - 2.034) *p*-value = <.0001	1.308* (1.046–1.634) *p*-value = 0.0183	1.412* (1.139–1.749) *p*-value = 0.0116
Gleason				
Unknown	46	1.275 (0.905–1.796) *p*-value = 0.1643	1.153 (0.798–1.667) *p*-value = 0.4483	
≤ 7	204	Reference	Reference	
≥ 8	231	1.266* (1.031–1.554) *p*-value = 0.0242	1.235 (0.997–1.529) *p*-value = 0.0532	
ECOG				
Unknown	202	1.658 (1.277–2.153) *p*-value = 0.0001	1.479 (1.124–1.947) *p*-value = 0.0052	1.538 (1.182–2.002) *p*-value = 0.0014
0	110	Reference	Reference	Reference
1	134	1.641* (1.240 - 2.171) *p*.-value = 0.0005	1.378* (1.024–1.855) *p*-value = 0.0343	1.447* (1.086–1.927) *p*-value = 0.0116
2/3/4	35	1.793* (1.179 - 2.727) *p*-value = 0.0063	1.558* (1.002–2.422) *p*-value = 0.0489	1.642* (1.074–2.510) *p*-value = 0.0219
Location of node metastases				
Unknown	315	0.913 (0.702–1.189) *p*-value =0.5002	0.880 (0.663–1.167) *p*-value = 0.3747	
Pelvic lymph node	80	Reference	Reference	
Iliac regional nodes	51	0.748 (0.512–1.094) *p*-value = 0.1346	0.763 (0.513–1.136) *p*-value = 0.1829	
Pelvic and iliac nodes	35	1.111 (0.734–1.681) *p*-value = 0.6187	1.110 (0.725–1.700) *p*-value = 0.6310	
Location of metastases				
Unknown	16	1.034 (0.525–2.034) *p*-values = 0.9232	1.085 (0.537–2.191) *p*-value = 0.8203	
Bone and lymph node metastases	429	1.092 (0.744–1.604) *p*-values = 0.6523	0.884 (0.589–1.326) *p*-value = 0.5509	
Visceral	36	Reference	Reference	

*Denotes statistically significance

^1^Univariate HR contains the results from fitting a univariate Cox model with each covariate of interest

^2^Multivariate (no selection HR) contains the results from fitting a multivariate model with all covariates of interest included, no selection procedure was applied

^3^Multivariate (backward selection HR) contains the results from fitting a multivariate model in which covariates with a *p*-value < 0.10 in the univariate approach are included. A backward selection procedure was applied. Only the results of the final model are displayed. The covariates considered include age, number of comorbidities, ALP, ECOG, Gleason, hemoglobin, PSA. Please note that although age was not significant at the 10% level, it was included as it is considered clinically important. Only ALP, hemoglobin, PSA and ECOG were retained in the final model. The other variables were removed as their *p*-values were > 0.10

However, in the multivariate analysis, LDH and Gleason score were no longer statistically associated with time to treatment failure. Only higher ALP, higher PSA and poorer ECOG PS scores were associated with shorter time to treatment failure, and higher hemoglobin with longer time to treatment failure. The same statistical significances were obtained in univariate and multivariate analysis of the relationship between clinical characteristics at initiation and PFS (data not shown).

It is important to notice that some of the unknown categories were statistically significant, which means that patients with missing values were different from those included in the reference category. According to HR for ECOG score, patients with missing values had lower time to treatment failure and PFS, given that the HR was close to one for ECOG PS scores of 2, 3 and 4.

When evaluating the baseline characteristics of those patients with longer time to treatment failure (ie, upper quartile) and the rest of the patients, several significant associations were identified. In the univariate regression analysis, patients with the longest time to treatment failure had either lower ALP (≤119 units/L; *p* < 0.001), or LDH (≤227 units/L; *p* = 0.0236), or PSA (≤56.2 ng/mL; *p* = 0.0004) levels than other patients; they were also more likely to have ECOG PS 0 (*p* = 0.0196) or to present with fewer bone metastases (*p* < 0.0001) when compared to patients with shorter time to treatment failure. Non-significant differences were found for Gleason score. Similar results were obtained in the multivariate regression analysis, apart from LDH that was found to be non-significant.

### Adverse drug reactions

Only 57 ADRs considered to be possibly related to AAP were reported from the source records. Thirty-nine patients (8.1%) had at least one ADR, of whom four had one or more serious ADRs. ADRs were given as the reason for treatment discontinuation for 17 patients (3.5%). The ADRs reported in three or more patients were hypertension (11 patients), oedema [6], asthenia [5], fatigue [4], nausea/vomiting [4], and abnormal enzyme levels [4].

## Discussion

This retrospective, non-interventional study provides insights into the characteristics, outcomes and subsequent treatment of 481 patients treated with AAP prior to chemotherapy in routine clinical practice in four European countries (Belgium, France, Germany, and the UK). It also contributes with evidence to our understanding of what patient characteristics may be driving better treatment response in this population when managed in the real-world.

The sample size for this study was larger than any other published real-world cohort in this setting [7, 8]. This avoids the uncertainty inherent in small samples, as evidenced by the narrow confidence interval around the median time to treatment failure. The multi-centre design across four countries captured a range of clinical settings, so the findings are likely to be generalizable within the countries studied and to similar European health systems.

The median time to treatment failure with AAP in our sample was 10.0 months (95% CI: 9.20–11.1), which was longer than in other real-world studies (5.3, 4.7 and 6.8 months) [7–9], but shorter than the treatment duration of 13.8 months seen in the phase III COU-AA-302 trial [1, 2]. Caution is needed when comparing the findings of this retrospective observational study with those of other studies because of differences in study design, patient assessment, and duration of follow-up, among other factors.

Firstly, the patient population in our real-world sample was slightly older than that reported in the phase III COU-AA-302 trial; had higher median PSA, ALP, and LDH levels at baseline; and included patients with ECOG status of 2 or 3 (all patients in the COU-AA-302 trial had ECOG status of 0 or 1) [1, 2]. Visceral metastases were present in 7.5% of patients in our sample, but were an exclusion criterion in the COU-AA-302 trial. Thus, our sample represented a real-world population not restricted by stringent eligibility criteria.

Secondly, a substantial proportion of patients (29.6% of those who discontinued) were reported to have stopped treatment with AAP as a consequence of PSA progression alone, with no other reason given. This is contrary to current clinical guidelines, which state that treatment for mCRPC should not be stopped for PSA progression alone; rather, at least two of the three progression criteria should be fulfilled before stopping treatment [6, 10]. One could speculate that time to treatment failure in this cohort might have been longer if PSA progression would have been confirmed by radiographic and/or clinical progression as per EAU-ESTRO-SIOG guidelines [10]. It is also possible that a lack of documentation in routine practice understated the frequency of clinical progression in the present study. Thirdly, 69 patients (14.3%) were still on treatment with AAP at the end of the data collection period, and were censored in the time to treatment failure and PFS analyses. As such, the study follow-up period did not allow for assessment of the study endpoints in the full sample of patients.

RECIST criteria were not used for the assessment of radiographic progression but instead imaging evidence of bone, CT and/or MRI scans were gathered. We found that a larger proportion of patients with radiographic progression had reported bone radiographic progression (70.1%), versus those with CT (55.2%) and/or MRI (7.8%). Given the higher number of bone metastases and the fact that these are considered non-evaluable by the RECIST criteria, we elucidate that the impact of not using RECIST in this study may have been relatively low.

Similar to time to treatment failure, the median PFS in our sample (10.8 months) was longer than that reported in other real-world studies (6.7 months) [7], but shorter than that reported in the COU-AA-302 trial (16.5 months) [1, 2]. As stated above, this may be partially explained by the fact that nearly 30% of patients discontinued AAP treatment due to PSA-only progression. The assessment of progression in this study was in line with clinical practice at the participating centre, rather than standardized criteria as in the clinical trial. Furthermore, the progression criteria used by the various physicians in our study may have varied. Local treatment protocols may not have been consistent with the most up-to-date treatment guidelines, and clinical practice may have evolved since some of the patients were treated. Similarly, the shorter PFS reported by Poon et al. [7] may be explained by several factors, including different patient characteristics, endpoint definitions and local clinical settings. In particular, older patients with more comorbidities at baseline were included in the Poon et al. study versus the current study (median age: 77 years vs 75 years; hypertension: 55.2% vs. 44.5%; diabetes: 27.6% vs. 14.8%). In addition, patients in the Poon et al. study presented a higher tumor burden than the patients in the current study (median baseline PSA: 212 ng/mL vs. 56.2 ng/mL) and a significantly higher proportion of patients with ECOG> 1 (37.9% vs. 7.3%).

Various baseline characteristics were found to influence time to treatment failure. Higher PSA, ALP, LDH, and Gleason score at diagnosis were all associated with shorter time to treatment failure in univariate analyses, as was poorer performance status. However, in multivariate analyses only higher PSA, higher ALP and poorer performance status retained this association. Normal levels of hemoglobin were associated with longer time to treatment failure in both analyses.

In line with other studies, these results hinted towards the possibility that certain clinical parameters may act as reliable predictors of treatment response. To further explore this hypothesis, a series of subgroup analyses were undertaken. Patients with 12 or more months between bilateral orchiectomy or ADT treatment and mCRPC diagnosis appeared to have longer time to treatment failure and longer time to disease progression than those with less than 12 months. These results were similar to those reported by Loriot et al. [4], who found that a shorter duration of response to ADT (< 12 months) was significantly associated to shorter PFS.

In addition, regression analyses showed that patients with either lower ALP (≤119 units/L), or lower LDH (≤227 units/L) or lower PSA values (≤56.2 ng/mL) or fewer bone metastases or better ECOG were more likely to be in the group with longest time to treatment failure (ie, upper quartile). Thus, these baseline characteristics may indeed play a role on response to AAP treatment. It is noteworthy that values for all of these characteristics were more favourable in the COU-AA-302 population than in the population of the current study, with the exception of haemoglobin and AST, which were not reported in the study publication for the clinical trial. However, to further validate this hypothesis, additional prospective research will be required.

AAP was well tolerated in this elderly cohort, with fewer than 2% of patients reported as discontinuing due to toxicity, and only 8% having an adverse drug reaction reported. However, the study was retrospective in design and adverse events were reported in the eCRF only when found in notes, thus, this needs to be considered when interpreting these results.

The retrospective design of the study may have imposed certain limitations. The study was not designed to properly evaluate survival, as there was no follow-up of patients who were still alive after the end of the data collection period (75% of patients remained alive one year after initiating AAP). Thus, the survival data from the study are difficult to interpret. The study population may also have been subject to selection bias against living patients. This is because eligible patients who were still alive had to give informed consent for their data to be used, and some may have refused or may have been lost to follow-up by the study centre. In contrast, all eligible deceased patients had their data used. In addition, some patients were still being treated with AAP at the end of data collection. Taken together, these factors may have led to underestimation of time to treatment failure and PFS.

It is worth noticing that the assessment of progression was based on information reported within the patients´ medical records as per physician’s evaluation, with no subsequent re-evaluations of the criteria as part of this study. The use of this retrospective real-world data may have resulted in certain limitations in terms of standardisation across different practices and settings given the lack of strict definitions as one would have for RCTs. However, stringent quality processes were put in place during the study execution to ensure quality and robustness of the data collected (eg, regular data monitoring, physicians´ query resolution and on-site source data verification).

The results from this non-interventional study provide deeper and valuable insights into the routine management and treatment of chemotherapy-naïve mCPRC patients in clinical practice conditions and can be used to complement other sources of evidence in this field, including data provided by RCTs and other non-interventional studies.

## Conclusions

The results of this retrospective, observational study provide valuable insights into the features, treatment and management of chemotherapy-naïve mCRPC patients treated with AAP in routine clinical practice. In real life, in older patients with more complex clinical presentation than those participating in the clinical trial, effectiveness and safety of AAP was maintained, despite presence of comorbidities and visceral disease.

## Additional file

Additional file 1: **Table S1.** Therapies following discontinuation with AAP. Table containing the first treatment prescribed to enrolled patients after discontinuation of AAP (DOCX 14 kb)

## Abbreviations

AAP: Abiraterone acetate plus prednisone
ADR: Adverse drug reaction
ADT: Androgen deprivation therapy
ALP: Alkaline phosphatase
ALT: Alanine aminotransferase
AST: Aspartate aminotransferase
CI: Confidence interval
CT: Computerized tomography
ECOG-PS: Eastern Cooperative Oncology Group Performance Status
eCRF: Electronic case report form
HR: Hazard ratio
IEC: Independent Ethics Committee
IQR: Inter-quartile range
IRB: Institutional Review Board
LDH: Lactate dehydrogenase
mCRPC: Metastatic castration-resistant prostate cancer
MRI: Magnetic resonance imaging
OS: Overall survival
PFS: Progression-free survival
PSA: Prostate-specific antigen
RCT: Randomised control trial
rPFS: Radiographic progression-free survival
SD: Standard deviation
UK: United Kingdom
