# Supplementary material for: Abiraterone acetate plus prednisone for the Management of Metastatic Castration-Resistant Prostate Cancer (mCRPC) without prior use of chemotherapy: report from a large, international, real-world retrospective cohort study
Source: BMC Cancer. 2019 Jan 14;19:60. doi: 10.1186/s12885-019-5280-6 (PMC6332550; doi:10.1186/s12885-019-5280-6)
Supplement: Supplementary file 1 — Table S1. Therapies following discontinuation with AAP. Table containing the first treatment prescribed to enrolled patients after discontinuation of AAP (DOCX 14 kb) [file 12885_2019_5280_MOESM1_ESM.docx]

**ADDITIONAL TABLES**

*Table A: Therapies following discontinuation with AAP*

|  | **Patients who discontinued AAP (N=412)** |
| --- | --- |
| **mCRCPC therapy** | |
|  | N (%) |
| No treatments prescribed | 151 (36.7) |
| Treatments prescribed | 261 (63.3) |
| Valid N | 412 |
| **First treatment after AAP discontinuation (N = 254 patients)*** | |
|  | N (%) |
| Chemotherapy/ Cytotoxic agent | 131 (51.6) |
| *Chemotherapy/ Cytotoxic agent - taxanes* | 129 (50.8) |
| *Chemotherapy/ Cytotoxic agent - others* | 2 (0.8) |
| Enzalutamide | 63 (24.8) |
| Hormonal therapy alone | 30 (11.8) |
| Radium-223 dichloride | 20 (7.9) |
| Investigational therapy | 1 (0.4) |

*please note that 7 patients from one site in the UK had missing data
